# In vitro gut microbial catabolism of ferulic acid is characterized by interindividual variability

**DOI:** 10.1038/s41538-026-00746-2

**Published:** 2026-02-16

**Authors:** Katerina Tomisova, Anna Mascellani Bergo, Veronika Jarosova, Ondrej Cinek, Lucie Hlinakova, Kateřina Valentová, Jaroslav Havlik

**Affiliations:** 1 Department of Food Science, Faculty of Agrobiology, Food and Natural Resources, Czech University of Life Sciences Prague, Prague, Czech Republic; 2 Department of Pediatrics, 2nd Faculty of Medicine, Charles University and University Hospital Motol, Prague, Czech Republic; 3 Institute of Microbiology of the Czech Academy of Sciences, Prague, Czech Republic

**Keywords:** Biochemistry, Microbiology

## Abstract

Interindividual variability in gut microbial metabolism of 4′-hydroxy-3′-methoxycinnamic acid (ferulic acid, FA), a major phenolic acid in cereals, may influence health outcomes and nutritional guidance; however, distinct metabotypes remain poorly defined. We investigated FA metabolism using in vitro fecal batch incubations from 18 individuals, combining ¹H NMR metabolomics with 16S rRNA amplicon sequencing. All individuals produced a shared set of FA-derived catabolites, but differed markedly in the rate and extent of FA conversion, enabling the identification of interindividual metabolic signatures. FA degradation was slower in older donors. Early 3-(3′-hydroxyphenyl)propanoic acid producers had lower tryptophan, and high 3-phenylpropanoic acid producers exhibited higher fumarate compared to higher 3-(3′,4′-dihydroxyphenyl)propanoic acid producers. Late 3′-hydroxyphenylacetic acid producers had higher alpha diversity. Microbiota composition showed only weak associations with metabolic signatures, suggesting that functional variability rather than taxonomic differences drives interindividual differences in FA catabolism. These findings support the existence of functional FA metabolic signatures and highlight the need for in vivo studies to elucidate their physiological relevance.

**Figure:**
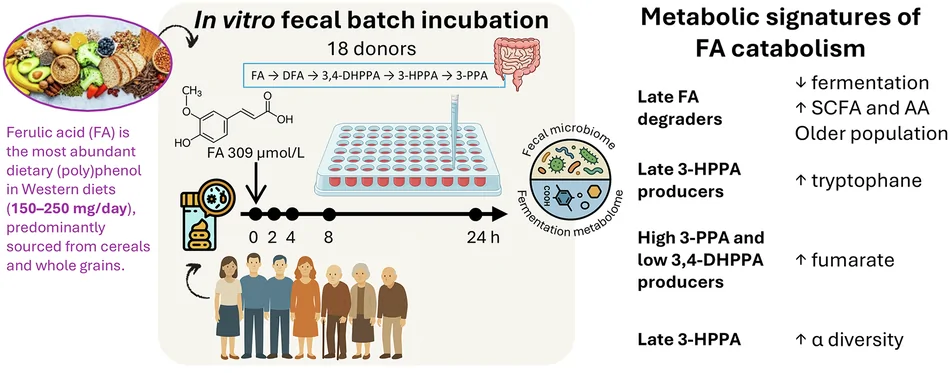

## Introduction

Plant-derived (poly)phenols, abundant in fruits, vegetables, coffee, and whole grains, are widely recognized for their health-promoting properties^1,2^. Interindividual variability in the metabolism of dietary bioactives presents a major challenge in nutritional research, as such differences can mask or attenuate true biological effects and hinder the reproducibility of findings, complicating the translation of research outcomes to individual health recommendations^3–5^. Nevertheless, these metabolic differences may hold the key to personalized nutrition^6^.

Following ingestion, (poly)phenols are only minimally absorbed in the upper digestive tract and undergo extensive catabolism by the colonic microbiota, producing a diverse set of bioactive derivatives that act locally in the gut and reach systemic circulation. Consequently, identical intakes of (poly)phenolic compounds may result in markedly different systemic exposures and biological responses across individuals^3^. This interindividual variation underpins the concept of metabotypes, defined as reproducible patterns of metabolite production observed in individuals exposed to the same compound or class of compounds^7^. Well-characterized examples include urolithin metabotypes that can derive from ellagitannins, equol from isoflavones, and enterolactone formation from lignans, all of which are tied to the presence/absence of specific taxa capable of unique metabolic transformation in the gut lumen^7^. Metabotypes can substantially influence biological responses, providing a basis for personalized nutrition strategies. Despite extensive research on certain (poly)phenols, metabotypes remain undefined for widely consumed compounds such as rutin or 4′-hydroxy-3′-methoxycinnamic acid (ferulic acid, FA).

FA is among the most abundant dietary phenolics, with an approximate intake in the Western diet of 150–250 mg/day, which could be much higher in coffee drinkers^8^. FA is the main phenolic compound in cereal grains, particularly wheat, where it may account for up to 90% of the total polyphenols; therefore, cereals represent the primary dietary source of FA, followed by coffee, fruits, and vegetables^9–12^. Due to limited absorption in the small intestine, up to 28% of ingested FA, depending on the food matrix, may reach the colon, where it becomes available for microbial catabolism^11,13^.

FA has been associated with neuroprotective, vascular, antioxidant, and anti-inflammatory effects^14–17^. These benefits are mediated not only by the parent compound, which is partly absorbed in the small intestine^18^, but also by gut-derived microbial catabolites resulting from colonic metabolism. The structure and bioactivity of these metabolites vary considerably among individuals^7,19^. While the microbial degradation of FA has been partially elucidated, its complete gut biotransformation remains incompletely characterized.

Once released from plant cell wall polymers by microbial esterases^20,21^, FA is typically hydrogenated to 3-(4′-hydroxy-3′-methoxyphenyl)propanoic acid (dihydroferulic acid, DFA), the predominant initial metabolite in fecal incubation models^22,23^. DFA is subsequently demethylated to 3-(3′,4′-dihydroxyphenyl)propanoic acid (3,4-DHPPA), dehydroxylated to 3-(3′-hydroxyphenyl)propanoic acid (3-HPPA), and further dehydroxylated to 3-phenylpropanoic acid (3-PPA). *α*- and *β*-oxidation of these intermediates can yield derivatives with shortened side chain^22–24^. Less frequent routes include the demethylation of FA to 3′,4′-dihydroxycinnamic acid (caffeic acid), conversion to 4′-hydroxycinnamic acid, and subsequent *α*-oxidation to 4′-hydroxyphenylacetic acid (4-HPAA)^25^. DFA and 3,4-DHPPA consistently dominate, highlighting their central role, while 3-HPPA and 3′-hydroxyphenylacetic acid (3-HPAA) appear mainly at later stages of microbial catabolism.

Additional products, such as homovanillic acid, 3′,4′-dihydroxyphenylacetic acid (3,4-DHPAA), caffeic acid, 4-hydroxy-3-methoxybenzoic acid (vanillic acid), and 4-vinylguaiacol, are occasionally detected but remain minor^22,26^. For example, 4′-hydroxy-3′-methoxyphenylacetic acid and 3,4-DHPAA can be derived from dimeric FA in dietary fiber^23^. Although microbial metabolism generates several aromatic acids, including phenylacetic derivatives, direct conversion of FA to phenylacetic acid (PAA) has not been consistently demonstrated.

Overall, the central pathway FA → DFA → 3,4-DHPPA → 3-HPPA → 3-PPA appears conserved, yet interindividual differences in metabolite abundance and dynamics highlight the influence of gut microbial diversity^22^. Among these metabolites, 3,4-DHPPA shows notable antioxidant, anti-inflammatory, and metabolic regulatory effects in cellular and animal models^27–29^. Its *para*-dehydroxylated derivative, 3-HPPA, exhibits weaker activity in immune modulation and gut–brain axis signaling^30,31^. In contrast, 3-PPA becomes the predominant metabolite during late-stage biotransformation and has been shown to have pronounced antimicrobial activity compared to its hydroxylated precursors^32^ while also promoting intestinal epithelial barrier function via aryl hydrocarbon receptor signaling^33^. Nevertheless, it is well established that key activities, such as the suppression of reactive oxygen species (ROS) and the inhibition of cytokine secretion by human peripheral blood mononuclear cells, decline in parallel with the decreasing hydroxylation of the aromatic ring^28,34^. Thus, interindividual variability in converting 3,4-DHPPA to 3-PPA may modulate FA-associated health effects.

Despite the widespread dietary presence of FA and known interindividual variation in microbial metabolism, distinct metabotypes of FA have not been described. This represents a key point in our understanding of how phenolic compounds are differentially metabolized in the gut and how this may influence their health effects. We hypothesized that interindividual differences in gut microbiota function give rise to distinct metabolic signatures of FA catabolism, reflected in differences in the timing and relative abundance of known microbial catabolites rather than in the formation of unique metabolites. Using an exploratory in vitro batch incubation approach, this study aimed to identify putative FA metabolic signatures and to examine their associations with gut microbiota composition. These findings may advance the understanding of dietary phenolics, support precision nutrition strategies, and provide a foundation for future validation in human trials.

## Results

### Overview of FA catabolites

**Fig. 1:**
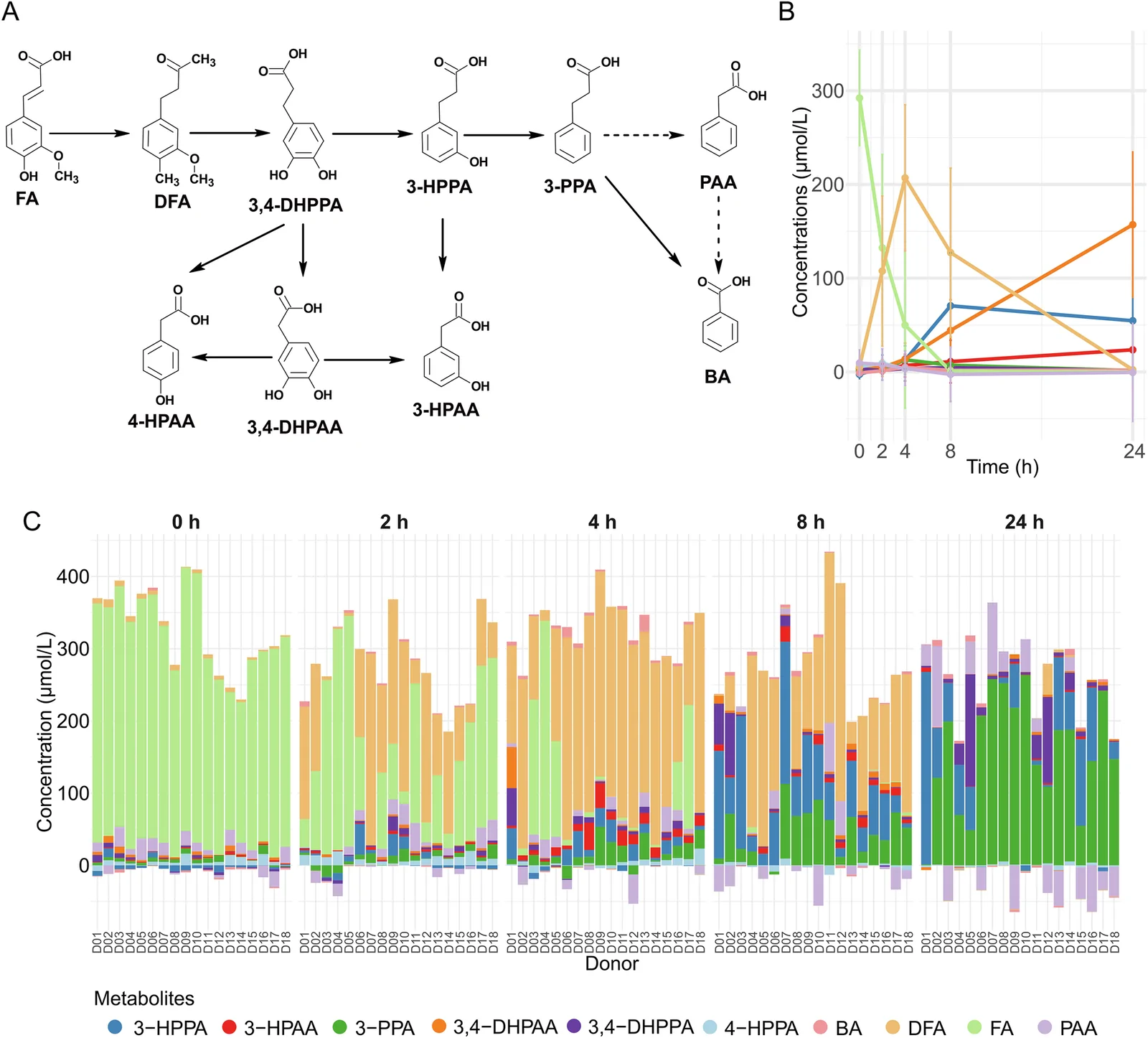
Temporal dynamics and interindividual variability in microbial ferulic acid catabolism. **A** Assumed degradation pathways, based on the appearance of catabolites observed in the study. Dashed arrows indicate quantified compounds for which there is a lack of evidence for direct conversion from ferulic acid. **B** Differential concentrations (FA-treated minus control) of FA and its microbial catabolites over a 24 h time course across all donors (*N* = 18). Data are presented as mean ± SD. Values are available in Supplementary Table S1. **C** Stacked bars showing interindividual variation in the concentration profiles of FA and its catabolites at 0, 2, 4, 8, and 24 h (FA-treated minus control). FA ferulic acid or 4′-hydroxy-3′-methoxycinnamic acid, DFA dihydroferulic acid or 3-(4′-hydroxy-3′-methoxyphenyl)propanoic acid, 3-HPPA 3-(3′-hydroxyphenyl)propanoic acid, 3-HPAA 3′-hydroxyphenylacetic acid, 3,4-DHPPA 3-(3′,4′-dihydroxyphenyl)propanoic acid, 3,4-DHPAA 3′,4′-dihydroxyphenylacetic acid, 3-PPA 3-phenylpropanoic acid, 4-HPAA 4′-hydroxyphenylacetic acid, BA benzoic acid, PAA phenylacetic acid.

To investigate the fate of FA degradation in the in vitro batch incubation system, FA and its microbial catabolites, including DFA, 3-HPPA, 3,4-DHPPA, 3,4-DHPAA, 3-HPAA, 3-PPA, 4-HPAA, PAA, and BA (Fig. 1A), were quantitatively monitored. Another major known and expected catabolite, 4-HPPA, could not be precisely quantified due to peak overlap and was therefore excluded; however, the estimated amounts were relatively minor. Interindividual variability in FA metabolism was assessed across 18 donors, with metabolite concentrations measured at five time points (0, 2, 4, 8, and 24 h) following the addition of FA in batch cultures (60 µg/mL, equal to 309 µmol/L). The temporal dynamics of FA degradation and downstream metabolite formation are illustrated in Fig. 1B, which presents the differential concentrations (FA-treated minus control) over a 24 h period. Mean/median concentrations (with SD or IQR) for FA and each FA catabolite at each time point are reported in Supplementary Table S1. Figure 1C highlights the substantial heterogeneity among individuals in both the extent and profile of microbial FA catabolism.

Immediately after FA addition (0 h), FA degradation began, and DFA emerged as a major early catabolite, peaking at 4 h (mean 292.27 µmol/L) and gradually declining due to further conversion. FA concentrations dropped sharply by 2 h, and it was fully catabolized by 8 h in most donors, although complete degradation occurred as early as 4 h in some cases. The extent of FA depletion and DFA accumulation varied considerably between donors, indicating marked interindividual differences in metabolic activity.

Among the intermediate catabolites, 3-HPPA and 3,4-DHPPA were detected only transiently, as they were formed and subsequently further catabolized. 3-HPPA appeared between 2 (mean 4.0 µmol/L) and 4 h (mean 12.48 µmol/L), while 3,4-DHPPA peaked early (4–8 h) in D03 and D04, and at 24 h in the remaining donors (mean 23.55 µmol/L). Other minor metabolites, including 4-HPPA, BA, 3,4-DHPAA, and 3-HPAA, exhibited only modest increases across the time points. 3-HPAA exhibited a transient profile with a maximum at 4 h or 8 h. 3,4-DHPAA was detected exclusively in high concentrations in one single donor, D03, at 4 h. 4-HPPA, which can arise from metabolic pathways other than FA and was also present in control samples, mainly produced between 2 and 4 h. Similarly, BA, one of the simplest low-molecular-weight phenolic catabolites of FA, also reported as a microbial degradation product of other phenolic compounds, showed interindividual variability, with possible peaks at 4 h or 24 h in some donors; however, its detection was limited by interference from control subtraction. In our experimental setup, PAA was detected but did not increase upon FA microbial catabolism, suggesting that it may not be derived from FA metabolism.

3-PPA began to accumulate at 4 h and became the predominant FA-derived product by 24 h. However, at this time point, several donors still showed detectable levels of intermediate precursors, such as 3-HPPA and 3,4-DHPPA, highlighting variability in the dynamics of FA catabolism.

As ^1^H NMR spectroscopy enables quantitative analysis, it provides an unbiased overview of the mass conversion of the original FA and its downstream catabolites. At 0 h, we detected 292.27 ± 51.70 µmol/L (mean ± SD) FA. At 2 h, we detected 273.91 ± 57.27 µmol/L of the total molar quantity of FA and its catabolites; at 4 h, 318.18 ± 40.34 µmol/L; at 8 h, 264.07 ± 65.27 µmol/L; and finally, at 24 h, 241.75 ± 66.01 µmol/L of the total moles of FA and its catabolites. Indeed, the converted mass concentration at 0 h corresponded to 56.75 ± 10.04 μg/mL (mean ± SD) FA. At 2 h, we detected 51.98 ± 10.45 μg/mL of the total mass of FA and its catabolites; at 4 h, 59.83 ± 6.88 μg/mL; at 8 h, 47.12 ± 11.71 μg/mL; and finally, at 24 h, 38.01 ± 9.75 μg/mL of the total mass of FA and its catabolites. Overall, these findings reveal distinct metabolic patterns and highlight the strong interindividual variability in microbial FA degradation, with distinct metabolic signatures of FA catabolism characterized by differences in the rate and extent of catabolite production.

### Defining FA metabolic signatures

**Fig. 2:**
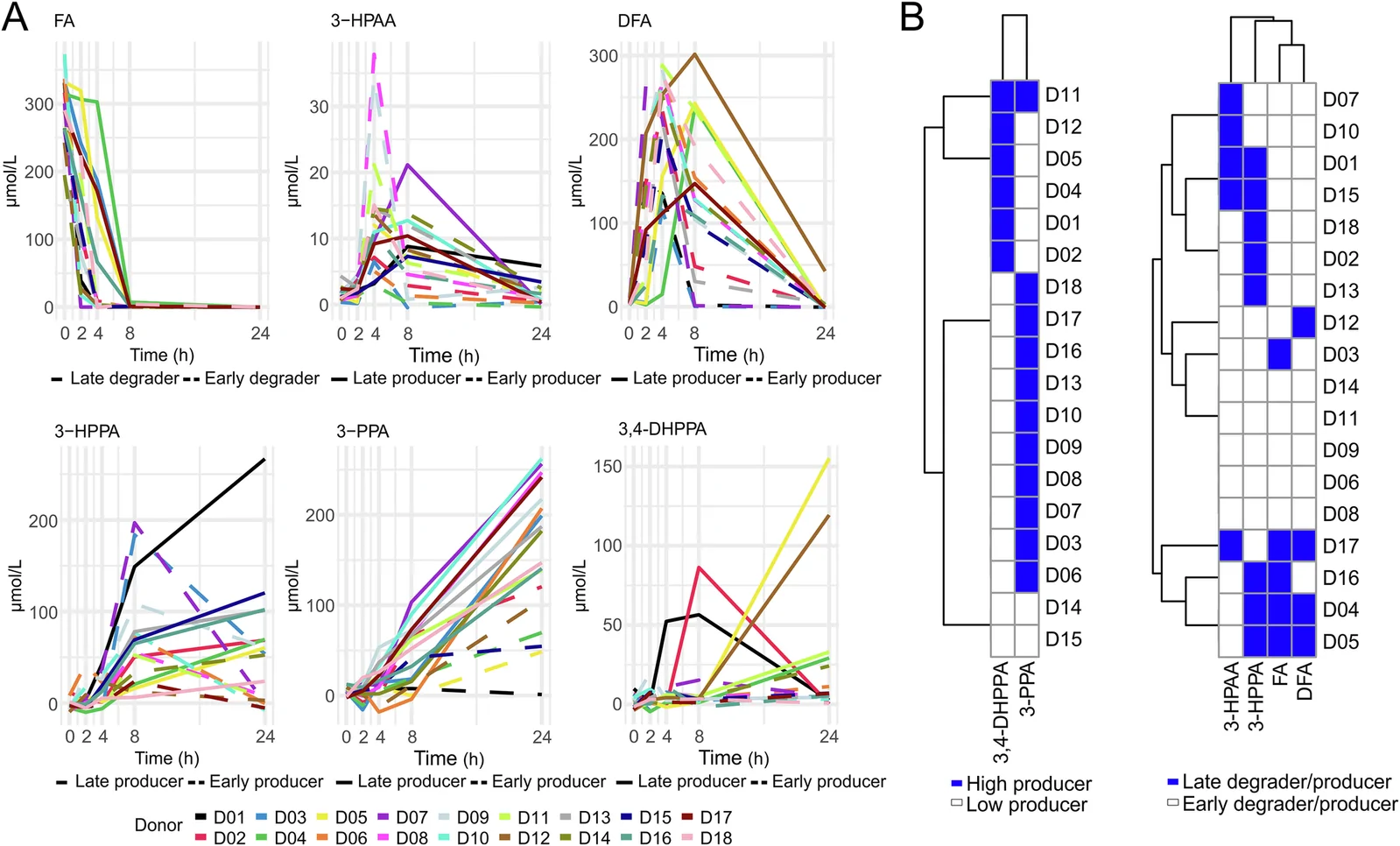
Individual-specific profiles of FA metabolites employed for the definition of individual metabolic signatures. **A** Differential concentrations (FA-treated minus control) of FA and its microbial catabolites over a 24 h time course for each donor (*N* = 18). **B** Heatmap showing metabolic signatures of FA catabolism from fecal inoculum from each of the 18 participants. Colors represent binary classification based on the criteria used to define donors, as described in Table 1, based on metabolite dynamics. FA ferulic acid or 4′-hydroxy-3′-methoxycinnamic acid, DFA dihydroferulic acid or 3-(4′-hydroxy-3′-methoxyphenyl)propanoic acid, 3-HPPA 3-(3′-hydroxyphenyl)propanoic acid, 3-HPAA 3′-hydroxyphenylacetic acid, 3,4-DHPPA 3-(3′,4′-dihydroxyphenyl)propanoic acid, 3-PPA 3-phenylpropanoic acid.

We were unable to identify unique catabolites produced exclusively by a subset of the donors in our study, which we could refer to as metabotypes. Instead, the same set of catabolites was observed across all donors. Time-course graphs were generated to show the production of each metabolite by fecal microbiota from individual donors (Fig. 2A). The production of these metabolites differed in timing of appearance (early or late), or marked differences in concentrations (high or low). Based on this behavior, binary classes describing metabolite dynamics were assigned (see Section 4.6), as described in Table 1. A heatmap was constructed to visualize patterns in these classes among donors (Fig. 2B), revealing an additional pattern that divided the population into high producers of 3-PPA and low producers of 3,4-DHPPA. Given the biological relevance of 3,4-DHPPA, particularly the preservation of its hydroxyl groups, which contribute to its antimicrobial properties, these groups were selected for further investigation into differences in microbial composition and metabolites reflecting microbial activity profiles (Fig. 2B).

**Table 1:** Criteria used to define binary classes for donors according to metabolite dynamics, considering both timing (early or late) and quantity (high or low)

	Definition based on time	
FA	Early degraders	Late degraders
FA	No presence at 4 h	Presence at 4 h
FA catabolite	Early producers	Late producers
DFA	Peak at 2 or 4 h	Peak at 8 h
3-HPPA	Peak at ≤ 8 h	Peak at 24 h
3-HPAA	Peak at 4 h	Peak at 8 h
	Definition based on concentration	
	High producers	Low producers
3-PPA	Peak ≥ 130 µmol/L	Peak < 130 µmol/L
3,4-DHPPA	Peak ≥ 30 µmol/L	Peak < 30 µmol/L

*FA* ferulic acid or 4′-hydroxy-3′-methoxycinnamic acid, *DFA* dihydroferulic acid or 3-(4′-hydroxy-3′-methoxyphenyl)propanoic acid, *3-HPPA* 3-(3′-hydroxyphenyl)propanoic acid, *3-HPAA* 3′-hydroxyphenylacetic acid, *3,4-DHPPA* 3-(3′,4′-dihydroxyphenyl)propanoic acid, *3-PPA* 3-phenylpropanoic acid.

This approach defined the five metabolic signatures investigated in the study: early (*N* = 13 (72.2%)) and late (*N* = 5 (27.8%)) degraders of FA; early (*N* = 14 (77.8%)) and late (*N* = 4 (22.2%)) producers of DFA. 3-HPAA (early, *N* = 13 (72.2%); late, *N* = 5 (27.8%)), 3-HPPA (early, *N* = 10 (55.6%); late, *N* = 8 (44.4%)), and a subset of donors categorized as high producers of 3-PPA while low producers of 3,4-DHPPA (*N* = 10 (55.6%) and vice versa (*N* = 5 (27.8%)).

To assess whether these metabolic signatures of FA catabolism were associated with demographic factors, we examined differences by sex, age, and BMI (Table 2). Among these, only FA degradation showed a significant association with age (*p* = 0.020): all individuals under 70 years were early degraders, while those over 70 were evenly distributed between early (*N* = 5, 50%) and late (*N* = 5, 50%) degraders.

**Table 2:** Demographic differences across each metabolic signature of FA catabolism defined in the study. Significant comparisons are highlighted with bold font

Metabolite	Age (years)			BMI z-score			Gender		
	Early degraders	Late degraders	*p*-value^a^	Early degraders	Late degraders	*p*-value^a^	Early degraders	Late degraders	*p*-value^b^
FA	47.4 ± 22.2	75.4 ± 2.7	**0.020**	24.6 ± 3.9	25.9 ± 1.1	0.336	M = 7, F = 6	M = 1, F = 4	0.233
	Early producers	Low producers	*p*-value^a^	Early producers	Low producers	*p*-value^a^	Early producers	Low producers	*p*-value^b^
DFA	53.0 ± 22.6	62.8 ± 21.2	0.456	24.9 ± 3.7	25.4 ± 1.6	0.878	M = 7, F = 7	M = 1, F = 3	0.353
3-HPPA	54.5 ± 22.3	12.0 ± 23.0	0.859	25.5 ± 3.3	25.8 ± 3.4	0.897	M = 4, F = 6	M = 4, F = 4	1.466
3-HPAA	55.5 ± 21.9	54.4 ± 24.6	0.621	25.6 ± 2.6	55.5 ± 21.9	0.503	M = 7, F = 6	M = 1, F = 4	0.233
	High 3,4-DHPPA producers	High 3-PPA producers	*p*-value^a^	High 3,4-DHPPA producers	High 3-PPA producers	*p*-value^a^	High 3,4-DHPPA producers	High 3-PPA producers	*p*-value^b^
3,4-DHPPA vs. 3-PPA	46.2 ± 25.7	63.9 ± 17.0	0.456	23.2 ± 3.6	25.1 ± 2.9	0.878	M = 3, F = 2	M = 5, F = 5	0.299

Data is presented as mean ± SD.

*FA* ferulic acid or 4′-hydroxy-3′-methoxycinnamic acid, *DFA* dihydroferulic acid or 3-(4′-hydroxy-3′-methoxyphenyl)propanoic acid, *3-HPPA* 3-(3′-hydroxyphenyl)propanoic acid, *3-HPAA* 3′-hydroxyphenylacetic acid, *3,4-DHPPA* 3-(3′,4′-dihydroxyphenyl)propanoic acid, *3-PPA* 3-phenylpropanoic acid, *M* male, *F* female.

^a^Wilcoxon test.

^b^Fisher’s exact test.

Further complexity was evident in the categorical heatmap (Fig. 2B), which showed that late FA degradation did not consistently correspond to delayed formation of its downstream metabolites. For instance, although donors D20, D06, D21, and D05 were all late FA degraders, their metabolite production patterns varied: D20 and D06 were late for 3-HPPA but early for 3-HPAA and, in the case of D20, also DFA; D21 was early for 3-HPPA but late for the others; and D05 showed early production of all three metabolites, DFA, 3-HPAA, and 3-HPPA (Fig. 2B). These findings indicate a lack of consistent alignment between FA degradation and metabolite formation, suggesting that distinct microbial taxa or functional redundancy may drive FA conversion along different biochemical pathways.

With our limited dataset, we were unable to establish metabolic signatures for 3,4-DHPAA, as only one donor in the dataset was a major producer. Similarly, we could not establish metabolic signatures for 4-HPPA, PAA, and BA, as they did not show clear category distinctions (Supplementary Fig. S1). We were also unable to define metabolic signatures for PAA, as it is typically produced from medium components even in control samples. The small proportion originating specifically as an FA metabolite had to be estimated by subtraction, which resulted in high variability and, in some cases, negative values.

### Associations between gut microbiota and FA metabolic signatures

To assess whether interindividual differences in FA metabolic signatures were associated with gut microbiota composition or overall microbial metabolic activity, fecal samples from all donors were analyzed by 16S rRNA gene sequencing and ^1^H NMR–based metabolomics.

**Fig. 3:**
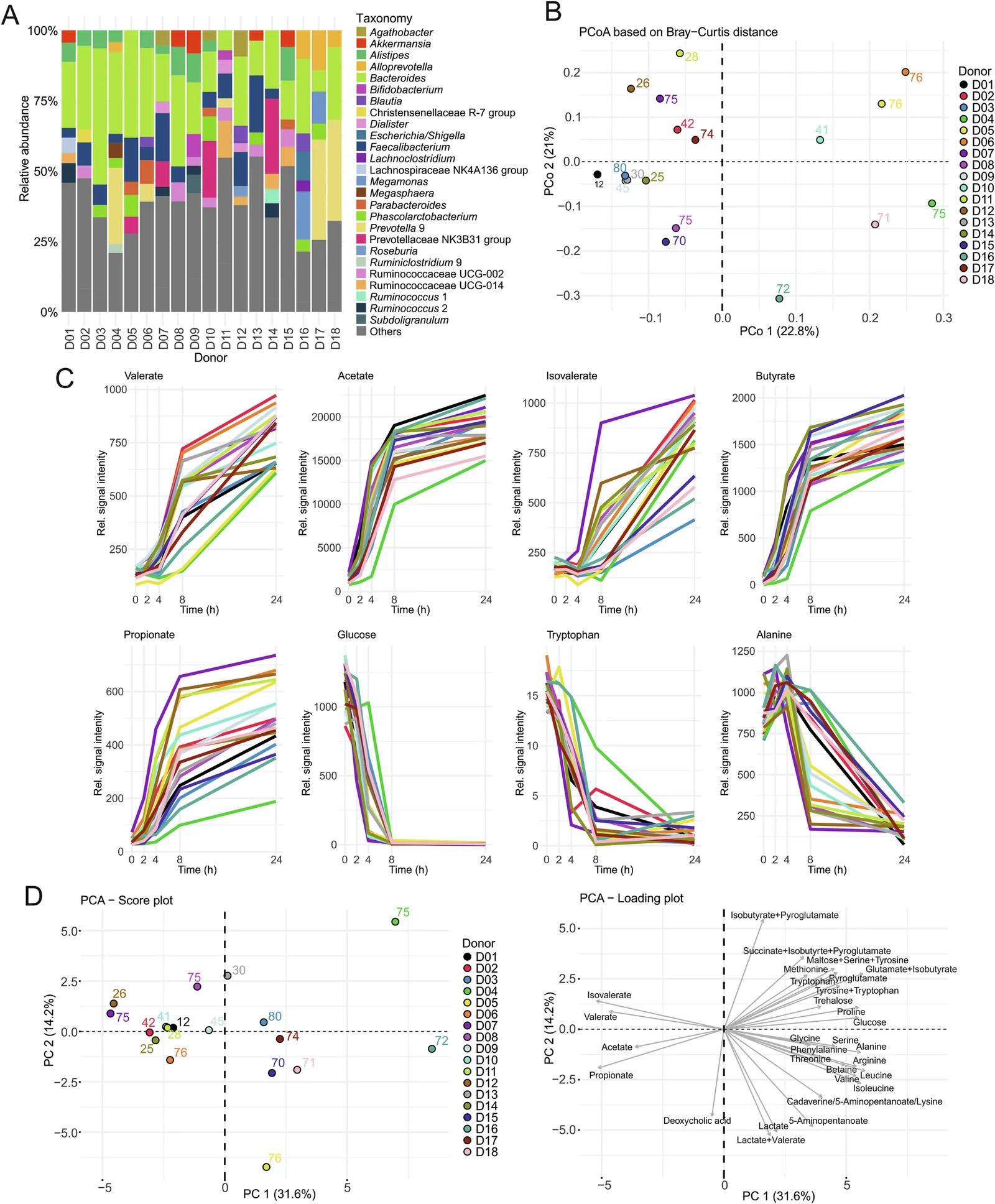
Interindividual variability in gut microbial composition and metabolic response. **A** Stacked bar plots showing the relative abundance of bacterial genera across fecal donors. **B** Principal coordinates analysis (PCoA) based on Bray–Curtis distances reveals interindividual differences in microbiota composition. Numbers next to the dots indicate the age of the corresponding donors. **C** Time-course profiles of selected microbial metabolites during in vitro microbial catabolism of ferulic acid across donors, showing dynamics in substrate utilization and metabolite formation. **D** Principal component analysis (PCA) biplot of metabolite profiles from in vitro fecal incubations, highlighting correlations between donors and key metabolites. Numbers above the dots indicate the age of the corresponding donors. For clarity, the biplot displays only the 30 variables with the highest loadings, while the PCA was performed using all variables.

At the phylum level, Bacteroidetes (44.89%) and Firmicutes (42.19%) predominated, followed by Proteobacteria (7.70%), Actinobacteria (2.16%), and Verrucomicrobia (1.55%) (Supplementary Fig. S2). At the genus level, Bacteroides was most abundant (26.33%), followed by Faecalibacterium (6.4%), Escherichia/Shigella (6.4%), and Prevotella (3.9%) (Fig. 3A). Principal coordinates analysis (PCoA; Bray–Curtis) indicated pronounced interindividual variability in microbiota composition (Fig. 3B). The first two axes (PCo1 and PCo2) explained 48% and 19% of the variance, respectively, without evident age-related clustering in this genus-level ordination.

To capture functional differences among donors, metabolites reflecting overall microbial activity, excluding FA and its direct catabolites, were quantified. The ^1^H NMR metabolomic analysis of the fermentation media revealed the presence of 54 microbial metabolites (Supplementary Table S2), including carbohydrates, amino acids, short-chain fatty acids (SCFAs), branched-chain fatty acids (BCFAs), and polyamines. Time-course profiles of the main microbial metabolites during in vitro biotransformation across donors, showing kinetics in nutrient utilization and metabolite formation, are shown in Fig. 3C. Temporal profiles were summarized as area under the curve (AUC) values over a 24 h period. Principal component analysis (PCA) of AUC values (Fig. 3D) showed substantial interindividual variation in microbial metabolic output, with PC1 and PC2 accounting for 31.6% and 14.2% of the variance, respectively. Sample separation was driven primarily by SCFAs (acetate, propionate) and BCFAs (isovalerate, valerate), which were more abundant in the younger population, suggesting reduced microbial metabolic activity in the older portion of the study population.

### Differences between early vs. late FA degraders

To examine the relationship between gut microbial composition and the microbial metabolome in early and late FA degraders, we compared alpha-diversity metrics, microbial genus composition, and metabolite profiles between the groups.

**Fig. 4:**
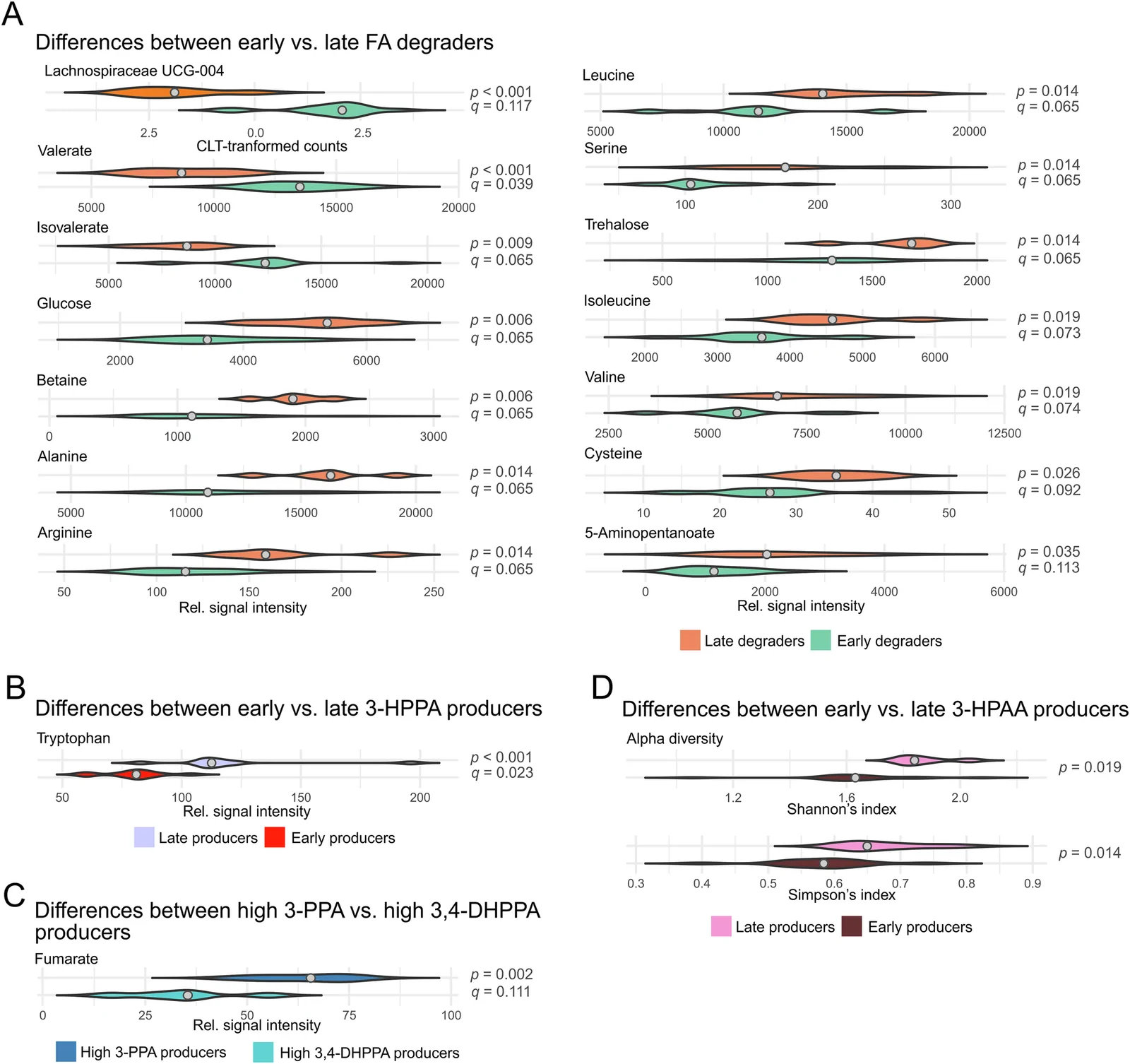
Changes in fecal microbiota and microbial metabolome for metabolic signatures of ferulic acid catabolism. **A** Differences between early and late ferulic acid degraders. **B** Differences between early and late 3-HPPA producers. **C** Differences between high 3-PPA and high 3,4-DHPPA producers. **D**Differences between early and late 3-HPAA producers. Significance was defined as *q* < 0.15. FA ferulic acid or 4′-hydroxy-3′-methoxycinnamic acid, 3-HPPA 3-(3′-hydroxyphenyl)propanoic acid, 3-HPAA 3′-hydroxyphenylacetic acid, 3,4-DHPPA 3-(3′,4′-dihydroxyphenyl)propanoic acid, 3-PPA 3-phenylpropanoic acid.

At the level of gut microbiota composition, only a limited number of differences were detected between early and late degraders. Early degraders exhibited a higher relative abundance of *Lachnospiraceae* UCG-004 (Fig. 4A), whereas no other taxa passed the predefined significance threshold. Alpha-diversity indices (Shannon and Simpson) did not differ significantly between groups, indicating that FA degradation rate was not associated with overall microbial diversity or broad compositional shifts.

In contrast, pronounced differences were observed in metabolites reflecting overall microbial metabolic activity (Fig. 4A). Early FA degraders had significantly higher AUC values for the branched-chain fatty acids (BCFAs) valerate and isovalerate, along with lower residual concentrations of several medium-derived substrates, including glucose, trehalose, and multiple amino acids (leucine, alanine, arginine, serine, valine, isoleucine, and cysteine). In addition, lower levels of betaine and 5-aminopentanoate were observed in early degraders. Together, these patterns indicate more rapid substrate utilization and increased proteolytic and fermentative activity in early FA degraders.

Notably, the differences observed in microbial metabolite profiles were substantially more pronounced than those detected in microbiota composition, which seems to be linked with the fact that FA degradation rate reflects a functional state of the microbial community rather than the presence of distinct taxonomic assemblages.

### Differences between early vs. late 3-HPPA producers

To assess whether variation in the timing of 3-HPPA formation was associated with differences in gut microbiota composition or microbial metabolic activity, we compared the microbial metabolome and gut microbiota composition between early and late 3-HPPA producers.

No significant differences in gut microbial composition were identified at the genus level between early and late producers, and neither Shannon’s nor Simpson’s diversity indices differed between groups, indicating that differences in 3-HPPA kinetics were not associated with broad microbiota structure. Among metabolites reflecting overall microbial activity, late 3-HPPA producers exhibited significantly higher tryptophan levels in the cultivation medium (Fig. 4B). As tryptophan was supplied by the medium, this indicates reduced microbial utilization of tryptophan in late producers, whereas early producers showed greater tryptophan utilization under identical conditions.

### Comparison of 3,4-DHPPA vs. 3-PPA producers

To further investigate interindividual variability in FA metabolism, we compared individuals classified as high 3,4-DHPPA producers with low 3-PPA levels to those with high 3-PPA and low 3,4-DHPPA levels, due to a strong inverse relationship between these compounds. Both share a three-carbon side chain linked to an aromatic ring, with 3-PPA featuring an unsubstituted phenyl ring and 3,4-DHPPA containing two hydroxyl groups. 3-PPA may arise from 3,4-DHPPA catabolism via dehydroxylation (Fig. 1A).

No significant differences in gut microbiota composition or alpha-diversity indices were detected between high 3,4-DHPPA and high 3-PPA producers.

Among metabolites reflecting microbial activity, fumarate levels were higher in high 3-PPA producers (Fig. 4C).

Given the role of fumarate as an intermediate in anaerobic microbial energy metabolism, this association suggests that differences in 3,4-DHPPA versus 3-PPA production may reflect variation in microbial metabolic state rather than differences in genus-level community composition, warranting further investigation in larger cohorts.

### Additional metabolic signatures of FA catabolism

Two additional metabolic signatures related to early and late producers of 3-HPAA or DFA were examined, which revealed less distinct differences in the microbial metabolite profiles and microbiota composition. For DFA, no significant differences were detected in either gut microbiota composition or microbial metabolome between early and late producers. Early producers of 3-HPAA demonstrated lower Shannon’s and Simpson’s diversity indices compared to late producers (Fig. 4D). However, no individual microbial taxa passed the significance threshold after correction for multiple testing, and no consistent differences were observed in microbial metabolite profiles.

## Discussion

This study presents the first systematic investigation of interindividual variability in FA microbial metabolism using a high-throughput in vitro fecal batch incubation model combined with ¹H NMR metabolomics and 16S rRNA gene–based microbiota profiling. Rather than identifying novel catabolites or unique metabolic products, we observed a conserved set of eight FA-derived metabolites across all donors, but with clear differences in timing and abundance that defined five distinct metabolic signatures. These mainly reflected early/late degradation of FA, early/late formation of DFA, 3-HPPA, or 3-HPAA, and high/low formation of 3-PPA, and 3,4-DHPPA. In contrast, BA, 3,4-DHPAA, PAA, and 4-HPAA showed no consistent individual patterns. Conceptually similar stratification based on microbial metabolic kinetics has been reported for catechin, where individuals were classified as fast or slow converters in an in vitro batch incubation model^35^.

The first signature distinguished early and late FA degraders: FA disappeared within 2 h in early degraders, while it persisted to 4 h in late degraders (Fig. 4A). Late degraders were significantly older, suggesting that age may be a factor in FA conversion to DFA. Age therefore emerged as a relevant factor influencing polyphenol catabolism, with slower conversion observed in older donors, consistent with our previous study on silymarin^36^. Aging has been associated with shifts in gut microbiota composition, including a reduced abundance of taxa such as *Bifidobacterium* and *Faecalibacterium*, and an increased prevalence of families like *Enterobacteriaceae*, which are often linked to proinflammatory states^37–39^. Aged gut microbiota exhibits reduced functional capacity for butyrate synthesis, without changes in taxa abundances^40^, which could also be the case for other metabolic pathways. While Shannon and Simpson diversity indices and overall microbiota composition were comparable between early and late degraders, apart from an increase in *Lachnospiraceae* UCG-004 for early degraders, the latter exhibited lower levels of BCFAs (isovalerate and valerate, products of BCAA catabolism) and higher amino acid concentrations, indicating age-related impairments in proteolytic activity. We did not observe significant differences in microbial alpha-diversity between early and late FA degraders, in contrast to observations in catechin metabolism, where faster conversion has been linked to higher alpha-diversity^35^. It should further be noted that FA concentrations at 0 h exhibited substantial variability (Fig. 1C). Given that identical amounts of FA were added to the incubation medium and samples were collected within minutes after homogenization by repeated pipetting, this variability likely reflects rapid interactions with the fecal matrix and surfaces, including adsorption phenomena and technical variability associated with fecal slurry handling.

DFA was the most abundant transient metabolite, yet no major microbiota or metabolome differences were observed between early and late DFA producers, potentially also due to class imbalance, as late producers were fewer in number and may not have provided sufficient statistical power.

3-HPPA was the second most abundant FA–derived metabolite. It is considered a valuable metabolic marker for the intake of certain types of (poly)phenols^41^. Subjects differed in early versus delayed production: early formation may occur proximally in the colon, increasing absorption, whereas late formation suggests distal metabolism. The hydroxyl group contributes to its bioactivity by acting as an electron donor, thereby enhancing its affinity for and activity toward various cellular and molecular targets^28,33,42,43^.

Delayed 3-HPPA producers exhibited reduced tryptophan catabolism, suggesting lower activity of microbial taxa involved in both pathways, and potentially shared metabolic routes. In the human colon, unabsorbed tryptophan is metabolized by gut microbiota into bioactive compounds such as indole, its derivatives, and tryptamine, which can affect host metabolism by enhancing glucagon-like peptide-1 (GLP-1) signaling or contributing to the gut–brain axis^44^. These metabolites, however, were not observable in the ¹H NMR-based metabolite profile used in this study.

Another key signature was the interindividual variation in the conversion of 3,4-DHPPA to 3-PPA. Because 3,4-DHPPA retains bioactive hydroxyl groups, interindividual differences at this step may impact the health effects of FA. Although present at lower levels than 3-PPA, 3,4-DHPPA is an antioxidant^45^ and anti-inflammatory^46^ metabolite and has been linked to metabolic benefits in cellular models. 3,4-DHPPA has also been shown to alleviate obesity, as well as regulate insulin resistance, lipid metabolism, and oxidative stress response in high-fat diet mice^29^. In our study, donors with high 3,4-DHPPA and low 3-PPA showed subtle differences in the microbial metabolic profiles, particularly in fumarate levels. These differences are more likely attributable to changes in microbial energy metabolism than to shifts in community composition, as 16S rDNA sequencing data showed no significant variation in taxon abundances. The presence of more fumarate in incubations with lower concentrations of 3,4-DHPPA remains unclear and requires further study. In anaerobic bacteria, fumarate is central as a terminal electron acceptor in anaerobic respiration pathways^47^. Fumarate concentrations in the medium may be influenced by the quality of anaerobic conditions, pH^48^, or the presence of microbial taxa capable of oxidizing succinate^49^ or utilizing fumarate for respiration, a pathway found in *E. coli* and certain Bacteroides species^48,50^.

An important aspect of this work is the integration of interindividual variability with a physiologically relevant experimental design. In the context of an exploratory in vitro biotransformation study, the inclusion of 18 fecal donors constitutes a robust sample size. Each donor represents a complex and independent microbial ecosystem, and the intentional inclusion of both sexes and a wide age range, together with a multi-timepoint design and integrated metabolomic and microbiota profiling, allowed us to capture interindividual differences relevant to FA catabolism. At the same time, the diet-relevant FA concentration used (309 µmol/L, equal to 60 µg/mL) reflects typical Western intake levels (150–250 mg/day) mainly from cereals and whole grains^8,9^ and approximates estimated caecal concentrations based on reported absorption rates^13^ and ileal flow volumes^51^, addressing the physiological relevance. Quantitative ¹H NMR metabolomics offered a robust and reproducible readout of metabolic fluxes, despite being less sensitive than mass spectrometry, enabling the detection of nine core microbial metabolites consistent with recent in vitro studies^22^. Trace-level compounds such as caffeic acid, vanillin, and homovanillic acid were not detected, likely due to their low abundance or rapid turnover^23,52^. The relative conservation of the total molar abundance of FA and its quantified catabolites, despite a more marked decline in total mass, indicates dominance of ring-retaining transformations yielding progressively lower–molecular-weight phenolic derivatives. The latter reduction in both molar and mass sums reflects a loss of FA-derived carbon from the quantified phenolic metabolite pool, potentially including conversion to CO₂, although this cannot be directly resolved by ^1^H NMR spectroscopy alone.

Nevertheless, several limitations must be acknowledged. The thresholds used to classify metabolic signatures (e.g., early vs. late and high vs. low) were chosen for practical reasons and were not intended as biologically validated cut-offs. Rather, they were used to structure the analysis of interindividual variation in FA catabolism. Statistical power to detect subtle associations between microbiota composition and biotransformations was limited, particularly for less frequent metabolic signatures. At this resolution, it is not possible to determine whether the non-observed variability in microbiota profiles reflects true biological characteristics or is primarily driven by the high interindividual variability typical for human fecal microbiota, which substantially reduces statistical power in differential abundance analyses. The limited ability to elucidate how interindividual differences in the microbiota drive the observed metabolic signatures is further compounded by the fact that microbiota profiling was performed exclusively on fecal inocula, providing a single, static snapshot of community composition. In contrast, FA catabolite profiles represent dynamic metabolic processes evolving over the course of the fermentation, limiting the interpretability of direct microbiota–metabolite associations. Moreover, reliance on 16S rDNA sequencing enabled characterization of community structure but did not capture functional potential or metabolic activity. Metagenomic approaches would be required to provide more robust functional insights into microbial pathways involved in FA metabolism. The batch fecal incubation system, although simple and reproducible, lacked pH control and host interactions, such as absorption or immune signaling, which limit direct extrapolation to in vivo conditions. However, its simplicity, reproducibility, and widespread use in the scientific community are notable advantages^53^. Furthermore, intermediate sampling between 8 and 24 h would help capture transient metabolites that may have been missed.

In conclusion, the primary aim of this study was to investigate whether distinct microbial metabolic signatures of FA catabolism exist for FA, a major dietary (poly)phenol widely present in whole grains, cereals, and plant-based foods. Using an in vitro fecal incubation model combined with quantitative ¹H NMR metabolomics and 16S rRNA gene–based microbiota profiling, we characterized the microbial catabolism of FA in samples from 18 healthy donors. However, the observed catabolism was continuous rather than discrete, with all donors producing the same core set of metabolites.

Five metabolic signatures of FA catabolism were defined based on early/late degradation or early/late and high/low production of key metabolites. FA degradation rate was significantly associated with age, with older donors (over 70 years old) more likely to be late degraders with delayed FA biotransformation. However, early FA degradation did not consistently align with early production of downstream metabolites. Microbiota profiling revealed no strong compositional differences among metabolic signatures, though early 3-HPAA producers showed higher microbial diversity. Metabolomic analysis revealed that tryptophan utilization was lower in late 3-HPPA producers and higher fumarate levels were found in high 3-PPA producers, compared to high 3,4-DHPPA producers.

Overall, our results demonstrate that FA microbial metabolism follows a continuous trajectory across individuals, yielding a conserved set of metabolites but distinct metabolic signatures. Age was associated with slower FA degradation, while variability in 3-HPPA and 3,4-DHPPA metabolism reflected differences in microbial function rather than community composition. These findings underscore the need to look beyond microbial diversity toward metabolic activity. Future studies should validate these metabolic signatures in vivo, with larger cohorts, longitudinal sampling, and controlled dietary interventions to assess their stability and physiological relevance. The integration of metabolomics, microbiota, and host data will be crucial for identifying microbial taxa and enzymes responsible and for clarifying their impact on health. Given FA abundance in cereals and whole grains, and its proposed role in the benefits of whole-grain diets, understanding microbiota-driven variability is essential. This knowledge could support the development of biomarkers for FA metabolism and contribute to personalized dietary strategies that aim to maximize the health benefits of whole-grain consumption. Lastly, an important contribution lies in demonstrating the utility of quantitative NMR spectroscopy, underscoring its advantages and robustness relative to other analytical methods within the applied study design.

## Methods

### Study design, subjects, and ethics statement

FA was incubated with human fecal microbiota under anaerobic conditions using a modified simple static fecal batch incubation system, based on 96-well deep-well plates, inoculated with feces from 18 healthy human donors. Samples of the incubation medium were collected at five time points (0, 2, 4, 8, and 24 h) for metabolite profiling by quantitative ^1^H NMR spectroscopy to identify FA and its catabolites, as well as to assess changes in the metabolome. Additionally, the fecal microbial composition for each donor was assessed using 16S rDNA sequencing.

The study included 18 healthy subjects (with no antibiotic use for at least 4 weeks prior to collection, no chronic or acute gastrointestinal diseases, and no dietary restrictions), comprising 8 females (44%) and 10 males (56%). Their ages ranged from 12 to 80 years (median: 70.5, IQR: 42.25), with a BMI z-score ranging from 16.22 to 31.07 (median: 24.73, IQR: 2.95). Human fecal samples were collected from volunteer subjects between April and December 2020 under habitual dietary conditions to preserve the gut microbiota in its ecologically relevant functional state; no (poly)phenol–free diet was imposed.

All subjects signed an informed consent form prior to participating in the study, ensuring their inclusion. The fecal collection for the study was conducted in accordance with the Declaration of Helsinki, and the protocol was approved by the Ethics Committee of the University Hospital Královské Vinohrady in Prague (Ref. No. LEK-VP/01/0/2019). Personal data was processed in accordance with the EU General Data Protection Regulation (GDPR).

### Chemicals

The chemicals used for the in vitro fecal batch incubation were obtained from Merck (Darmstadt, Germany) and stored according to the manufacturer’s specifications. Calcium chloride (CaCl₂, ≥96%), manganese chloride tetrahydrate (MnCl₂·4H₂O, ≥98%), cobalt chloride hexahydrate (CoCl₂·6H₂O, ≥98%), ferric chloride (FeCl₃, ≥97%), ammonium carbonate (NH₄CO₃, ≥99%), sodium carbonate (Na₂CO₃, ≥99%), disodium hydrogen phosphate (Na₂HPO₄, ≥98%), potassium dihydrogen phosphate (KH₂PO₄, ≥99%), magnesium sulfate (MgSO₄, ≥98%), cysteine hydrochloride ( ≥ 98%), sodium sulfide (Na₂S, ≥97%), sodium hydroxide (NaOH, 1 M solution), vitamin K₁ and hemin. The fermentation medium, sodium phosphate buffer, and reducing solution were stored at 4 °C for up to one month before use. 4′-Hydroxy-3′-methoxycinnamic acid ( ≥ 98%, ferulic acid, FA), sodium azide ( ≥ 99.5%), and dimethylsulfoxide (DMSO) were purchased from Sigma-Aldrich, Prague, Czech Republic. All chemicals and reagents used for ^1^H NMR spectroscopy analysis were of analytical grade. Dipotassium phosphate (99%, K_2_HPO_4_), disodium hydrogen phosphate (99%, Na_2_HPO_4_), deuterium oxide (99.9%, D_2_O), and phosphoric acid ( ≥ 85 wt.% in H_2_O, H_3_PO_4_) for phosphate buffer preparation were purchased from VWR (Radnor, PA, USA). The 3-(trimethylsilyl)propionic-2,2,3,3-*d*_*4*_ acid sodium salt (99%, TSP) was purchased from Sigma-Aldrich (St. Louis, MO, USA).

### In vitro fecal batch incubation system

A static, high-throughput in vitro fecal batch incubation system was implemented using 96-well deep-well plates (well capacity 2.0 mL). Details of the system setup, including the composition of the incubation medium, reducing solution, and buffers, have been reported previously^36^ and the protocol was adapted from Havlik et al., 2020^54^. All donors independently collected fecal samples using a standardized collection kit. Samples were deposited into a 1 L plastic container (FECOTAINER, Excretas Medical, Enschede, NL) lined with a plastic bag. Following sample deposition, an oxygen-absorbing AnaeroGen sachet (Oxoid, Thermo Fisher Scientific, Basingstoke, UK) was placed inside the bag, which was then closed around the sample, and the container was sealed with its lid. Samples were kept at ambient temperature and transferred to the laboratory within 2 h of collection. The fecal bulk was homogenized for 30 s in a stomacher bag (Stomacher 400 Circulator, Laboratory Blender, EU) with sodium phosphate buffer, and the resulting 24% (w/v) fecal slurry was filtered through a nylon mesh.

The FA stock solution (10 mg/mL) was prepared in DMSO and stored at 4 °C. A working solution was prepared by diluting the FA stock solution in the fermentation medium and added to the incubations, resulting in a final FA concentration of 60 µg/mL corresponding to 309 µmol/L (with a final DMSO concentration of 0.5% v/v).

Incubations were performed in wells containing 835 µL of fermentation medium and 40 µL of reducing solution. Plates were placed in vacuum-sealed bags together with an AnaeroGen sachet and stored at 4 °C overnight. Prior to inoculation, plates were equilibrated to 37 °C, after which 100 µL of fecal slurry and 25 µL of FA working solution were added, resulting in a final working volume of 1.0 mL per well.

The fermentation system comprised multiple identically designed plates. Two types of controls were included: in positive controls, fecal slurry was replaced with phosphate buffer to assess FA stability in the absence of microbial activity; in negative controls, also referred to as controls, the FA working solution was replaced with DMSO while fecal slurry was retained. As FA showed no appreciable degradation under control conditions, data from positive controls were not included in subsequent analyses. Separate plates were used for each time point and incubated under the same conditions as described above. Samples (950 µL) were collected at 0, 2, 4, 8, and 24 h, and were immediately mixed with sodium azide (50 µL) to arrest microbial activity. The 0 h time point represents the earliest practically achievable sampling immediately following mixing of FA with the fecal slurry. Fermentation samples were stored at −80 °C until analysis.

To reduce intra-assay variability, experiments were conducted over multiple days as independent experimental batches. On each day, incubations were performed using fecal samples from two individual donors, each processed independently. Donors were selected randomly, irrespective of sex or age.

Anaerobic conditions were established using a reducing solution, displacement of oxygen by flushing the medium with oxygen-free nitrogen, anaerobic generator sachets, and overnight pre-equilibration of sealed plates prior to incubation. Anaerobiosis at the start of the experiment was verified using phenol red as a redox indicator. No pH control was applied during incubation; buffering capacity was provided solely by the medium, and the initial pH was adjusted to 7.0 prior to inoculation.

### Microbial metabolome and quantification of FA and its catabolites

Sample preparation and data acquisition were carried out as described by Tomisova et al. (2024)^37^. Samples were prepared and measured in a random order. Spectra were acquired on a Bruker Avance III HD 500.23 MHz spectrometer using a BBFO probe (Bruker BioSpin GmbH, Ettlingen, Germany) with a setting as described in Tomisova et al. (2024)^37^. Spectra quality was assessed based on the symmetry of the TSP signal and a width of less than 1 Hz.

For the metabolome investigation, the spectra were manually phased, while baseline correction and binning were performed using an in-house script in MATLAB R2022a (MathWorks, Natick, MA, USA). Baseline correction involved a multipoint baseline correction in user-defined segments, ensuring consistent pre-processing for all spectra. Spectra between δ_H_ 0.5 and 9.0 ppm (excluding the residual water region, δ 4.7–5.1 ppm) were reduced into defined buckets; each bin representing a spin system or a part of a spin system that was ideally pure, distinct, and quantitative—one bin for each metabolite. The bins’ ranges were chosen after annotating superimposed spectra (Supplementary Table S2) using 2D NMR spectra and annotating a subset of spectra with the Chenomx NMR Suite version 9.0 (Chenomx, Edmonton, AB, Canada), which included both the built-in reference library and our in-house database. For statistical processing, buckets were normalized to the area of the internal standard TSP. In addition, the area under the curve (AUC) values for each metabolite over 24 h were calculated using the trapezoidal method.

FA and its catabolites, including DFA, 3-HPPA, 3,4-DHPPA, 3,4-DHPAA, 3-HPAA, 3-PPA, 4-HPPA, 4-HPAA, PAA, and BA, were quantified using Chenomx NMR Suite version 9.0, with precise peak fitting referenced to the internal chemical shift standard TSP. Metabolite annotations and quantifications were performed using an in-house spectral library constructed from authentic standards, all of which were obtained from VWR (Avantor, CZ). The selection of this metabolite panel was guided by prior reports on FA catabolism and anticipated pathways of FA biotransformation^22,55^. We also compared the spectra of control samples with those from FA-supplemented wells to identify any spectral changes indicative of previously uncharacterized metabolites; however, no such additional metabolites were detected. The FID signals were processed in MestReNova software (Version 14.1.0; Mestrelab Research S.L., Santiago de Compostela, Spain), and the ^1^H NMR spectra were manually phased, baseline-corrected using the Whitaker smoother algorithm, and the TSP signal was referenced at 0.0 ppm. The pre-processed spectra were then exported as JCAMP files and imported into Chenomx NMR Suite. Finally, the concentrations were exported and adjusted for dilutions and expressed as µmol/L of incubation medium.

### Microbiota profiling

Microbiota analysis was conducted as described in Cinek et al. (2018)^56^ on an aliquot of the collected feces stored at −80 °C until analysis. Total DNA for amplicon sequencing was extracted from unprocessed stool samples, with DNeasy PowerSoil Kit (Qiagen, Hilden, Germany) according to the manufacturer’s instructions. Once the quantity of DNA was accessed, the V4 region of the 16S rRNA gene was amplified with specific primers described by Kozich et al. (2013)^57^. The PCR reaction contained 18 µL AccuPrime™ Pfx SuperMix (Invitrogen, Carlsbad, CA), 1.2 µL of each primer (10 µM) and 1.2 µL of extracted DNA. The cycling conditions were as follows: initial denaturation at 95 °C for 5 min, 30 cycles of 95 °C for 15 s, 55 °C for 30 s, and 68 °C for 1 min, followed by a final step at 68 °C for 5 min. The amplified indexed DNA fragments were purified and brought to the same concentration using the SequalPrep™ Normalization Plate Kit (Applied Biosystems™, Waltham, MA). Each sample was amplified in duplicate. The libraries were balanced and sequenced on the MiSeq platform (Illumina, San Diego, CA, USA) using the V2 kit of 2 × 250 bp reads.

The raw data were downloaded as demultiplexed *fastq* files with trimmed adapters. After quality inspection, data were processed in the DADA2 pipeline^58^. Reads were filtered with maximum allowed number of expected error equal to 2, demultiplexed, chimerae removed, and the size distribution of amplicons checked. The amplicon sequence variants (ASV) were counted and taxonomically classified using the SILVA reference database^59^, version 132. Negative controls were checked for the absence of a significant signal, and the mock community positions were assessed for agreement with their declared content. To verify agreement between replicates, an ordination plot was created by principal coordinate analysis of the Bray–Curtis dissimilarity among individual reactions; after its inspection, duplicates were merged by summing counts. For downstream analysis, taxonomic agglomeration was performed at various levels (phylum, class, order, family, genus, and amplicon sequence variant [ASV]). At the phylum level, the ratio between Bacteroides and Firmicutes was calculated. Taxa present in fewer than 20% of samples were excluded. CLR transformation was applied to non-rarefied data.

### Donor classification based on metabolite dynamics

Donors were classified based into binary classes describing metabolite dynamics, specifically the disappearance of FA or the production of metabolites, considering timing of appearance (early or late) and quantity (high or low) (Table 1). These criteria were selected following an initial exploratory assessment of the dataset, during which consistent trends became evident in the graphical outputs and were defined as metabolic signatures. The decision, while heuristic in nature, was motivated by the need to reduce data dimensionality and to define simple dynamic parameters suitable for subsequent evaluation. Some of the quantified catabolites were not included in Table 1 as they were considered not varying enough for the definition of metabolic signatures. A heatmap was generated in R using *pheatmap* v 1.0.12^60^ using binary classes according to Table 1 to visualize patterns in the production of multiple metabolites. This heatmap has led to an identification of a pattern of one additional metabolic signature. Each of the described metabolic was further used for statistical analysis.

### Statistical analysis

All statistical analyses were performed in Rstudio (R v. 4.3.3)^61^. FA and FA-catabolite concentrations were calculated by subtracting metabolite levels measured in donor-matched negative control wells from those in the corresponding FA-treated wells (FA-treated minus control). This correction was applied individually for each donor to account for baseline metabolite production arising from medium components other than FA. Summary statistics (mean, standard deviation, coefficient of variation, median, and interquartile range) were computed. To visualize the relative contribution of individual metabolites to the overall metabolic response, stacked bar plots were generated from FA-treated minus control data at each time point.

Statistical analyses were performed to assess potential differences in demographic characteristics. Differences in age and BMI were evaluated using the Wilcoxon test, while differences in sex distribution were assessed using Fisher’s exact test. These analyses aimed to determine whether demographic factors influenced the classification groups and to ensure that these variables did not confound observed patterns.

Alpha (within-sample) diversity was assessed using Shannon’s and Simpson’s indices on rarefied data (sequencing depth of 14,284 reads per sample). Wilcoxon tests from base R were used to evaluate differences in alpha diversity. Similarly, Wilcoxon tests were used to evaluate CLR-transformed taxonomy (at all levels) and microbial metabolome dynamics, as described in Section 2.6. *P*-values were corrected for false discovery rate (FDR) using the Benjamini-Hochberg method (*q*-values). Principal Coordinates Analysis (PCoA) was performed to explore patterns of similarity among samples based on their multivariate profiles. A distance matrix was first computed using the Bray–Curtis, and PCoA was then applied to reduce dimensionality while preserving the relative dissimilarities between samples using *vegan* package v 2.6-10^62^. The resulting coordinates were used to visualize sample clustering and assess the separation between groups.

Principal Component Analysis (PCA) was performed to reduce the dimensionality of the microbial metabolome, based on the calculated AUC, and to identify the major sources of variation among samples. Data were centered and autoscaled prior to analysis to ensure comparability across variables. PCA was performed using the prcomp function from base R, and the resulting principal components were utilized to visualize the sample distribution and investigate patterns of variation. The proportion of variance explained by each component was examined to assess its relative importance. The microbial metabolome was analyzed using Wilcoxon tests to assess differences in the calculated AUC of each metabolite among donors with different FA metabolite dynamics, as described in Section 2.6. *P*-values were corrected for FDR using the Benjamini-Hochberg method (*q*-values).

In this study, we applied an FDR threshold of *q* < 0.15 to identify features of interest, reflecting the exploratory nature of our analysis and the aim to capture suggestive associations for further validation.

All plots were generated using ggplot2 v. 3.5.0^63^, with custom themes for clarity and consistency.

## Supplementary information

41538_2026_746_MOESM1_ESM.

## Data Availability

The raw 16S sequencing data, along with relevant metadata, have been deposited in the NCBI SRA under project number PRJNA1399167. The metabolomics data, along with relevant metadata, have been deposited to the Zenodo data repository: 10.5281/zenodo.18197227.
