# Supplementary material for: In vitro gut microbial catabolism of ferulic acid is characterized by interindividual variability
Source: NPJ Sci Food. 2026 Feb 16;10:71. doi: 10.1038/s41538-026-00746-2 (PMC12921213; doi:10.1038/s41538-026-00746-2)
Supplement: Supplementary file 1 [file 41538_2026_746_MOESM1_ESM.docx]

**Supplementary Material**

***In Vitro* Gut Microbial Catabolism of Ferulic Acid is Characterized by Interindividual Variability**

Katerina Tomisova, Anna Mascellani Bergo, Veronika Jarosova, Ondrej Cinek, Lucie Hlinakova, Kateřina Valentová and Jaroslav Havlik*

*Corresponding author: Tel: +420 224382965; E-mail: havlik@af.czu.cz (J. Havlik)

**Table of contents**

|  |  | Page |
| --- | --- | --- |
| **Supplementary Table S1** | Mean/median concentrations (with SD or IQR) and coefficients of variation (CV) for FA and each catabolite at each time point. | 2 |
| **Supplementary Table S2** | List of bins applied in the study | 3 |
| **Supplementary Figure** **S1** | Differential concentrations (FA-treated minus control) of FA microbial catabolites over a 24 h time course for each donor (N = 18) | 5 |
| **Supplementary Figure S2** | Stacked bar plots showing the relative abundance of bacterial phyla across fecal donors. | 5 |

**Supplementary Table S1** Mean/median concentrations (with SD or IQR) and coefficients of variation (CV) for FA and each catabolite at each time point. Analytical variability was assessed using the coefficient of variation (CV), calculated as the standard deviation divided by the mean and expressed as a percentage. FA: ferulic acid, 4′-hydroxy-3′-methoxycinnamic acid; DFA: dihydroferulic acid, 3-(4′-hydroxy-3′-methoxyphenyl)propanoic acid; 3-HPPA: 3-(3′-hydroxyphenyl)propanoic acid ; 3-HPAA: 3′-hydroxyphenylacetic acid; 3,4-DHPPA: 3-(3′,4′-dihydroxyphenyl)propanoic acid; 3,4-DHPAA: 3′,4′-dihydroxyphenylacetic acid; 3-PPA: 3-phenylpropanoic acid; 4-HPAA: 4′-hydroxyphenylacetic acid; BA: benzoic acid; PAA: phenylacetic acid.

| **Metabolite** | **Time point** | Median (IQR) (µmol/L) | Mean ± SD (µmol/L) | CV (%) |
| --- | --- | --- | --- | --- |
| FA | 0 h | 295.5 (263.52 – 331.69) | 292.27 ± 51.71 | 17.69 |
| FA | 2 h | 107.55 (48.75 – 216.37) | 132.3 ± 99.61 | 75.29 |
| FA | 4 h | 4.01 (0.08 – 52.34) | 49.91 ± 88.58 | 177.49 |
| FA | 8 h | 0.08 (-0.05 – 0.80) | 0.86 ± 1.94 | 224.36 |
| FA | 24 h | -0.08 (-0.16 – 0.04) | -0.04 ± 0.17 | -430.8 |
| DFA | 0 h | 5.68 (3.56 – 7.27) | 5.50 ± 2.50 | 45.53 |
| DFA | 2 h | 106.45 (36.84 – 153.91) | 107.59 ± 80.03 | 74.38 |
| DFA | 4 h | 229.79 (140.41 – 269.97) | 207.03 ± 78.03 | 37.69 |
| DFA | 8 h | 127.89 (60.97 – 182.21) | 127.31 ± 90.09 | 70.76 |
| DFA | 24 h | -0.59 (-0.76 – -0.19) | 2.26 ± 10.12 | 448.52 |
| 3-HPPA | 0 h | -3.28 (-5.42 – -2.29) | -2.65 ± 5.42 | -204.69 |
| 3-HPPA | 2 h | -0.04 (-3.65 – 6.82) | 4.6 ± 13.36 | 290.64 |
| 3-HPPA | 4 h | 10.96 (1.34 – 22.7) | 12.48 ± 15.41 | 123.5 |
| 3-HPPA | 8 h | 59.95 (26.11 – 77.65) | 70.52 ± 56.39 | 79.96 |
| 3-HPPA | 24 h | 53.05 (3.80 – 69.34) | 54.59 ± 66.79 | 122.34 |
| 3-HPAA | 0 | 1.3 (0.97 – 1.77) | 1.50 ± 0.94 | 62.45 |
| 3-HPAA | 2 | 2.18 (1.46 – 2.78) | 2.21 ± 1.19 | 53.77 |
| 3-HPAA | 4 | 10.91 (6.69 – 14.55) | 12.90 ± 9.74 | 75.51 |
| 3-HPAA | 8 | 6.81 (3.32 – 10) | 7.13 ± 5.56 | 77.90 |
| 3-HPAA | 24 | 0.91 (0.43 – 2.06) | 1.45 ± 1.50 | 103.48 |
| 3,4-DHPPA | 0 h | 0.13 (-1.67 – 2.96) | 1.04 ± 3.75 | 358.80 |
| 3,4-DHPPA | 2 h | 1.65 (0.81 – 3.76) | 3.11 ± 5.45 | 175.39 |
| 3,4-DHPPA | 4 h | 3.67 (1.23 – 7.75) | 6.78 ± 12.10 | 178.59 |
| 3,4-DHPPA | 8 h | 3.32 (1.89 – 4.61) | 10.99 ± 22.81 | 207.51 |
| 3,4-DHPPA | 24 h | 6.08 (4.48 – 20.93) | 23.55 ± 42.90 | 182.14 |
| 3,4-DHPAA | 0 h | 2.76 (0.92 – 4.11) | 3.2 ± 3.01 | 94.14 |
| 3,4-DHPAA | 2 h | 1.23 (-0.15 – 2.99) | 1.51 ± 1.76 | 117.00 |
| 3,4-DHPAA | 4 h | 0.31 (-0.44 – 1.42) | 3.61 ± 13.34 | 369.14 |
| 3,4-DHPAA | 8 h | 4.00 (1.86 – 6.65) | 4.61 ± 3.08 | 66.87 |
| 3,4-DHPAA | 24 h | 1.41 (0.09 – 3.14) | 1.52 ± 2.40 | 158.16 |
| 3-PPA | 0 h | 4.12 (1.33 – 5.69) | 4.09 ± 3.63 | 88.88 |
| 3-PPA | 2 h | 5.36 (0.98 – 9.06) | 4.39 ± 9.10 | 207.30 |
| 3-PPA | 4 h | 10.17 (5.51 – 22.53) | 13.54 ± 17.19 | 126.95 |
| 3-PPA | 8 h | 46.9 (16.27 – 67.83) | 44.26 ± 32.70 | 73.88 |
| 3-PPA | 24 h | 164.73 (109.69 – 214.87) | 157.08 ± 78.42 | 49.92 |
| 4-HPPA | 0 h | 4.69 (2.80 – 6.76) | 5.43 ± 5.96 | 109.70 |
| 4-HPPA | 2 h | 8.87 (5.29 – 12.33) | 9.25 ± 5.33 | 57.63 |
| 4-HPPA | 4 h | 3.54 (-1.28 – 6.88) | 3.36 ± 7.27 | 216.53 |
| 4-HPPA | 8 h | -0.16 (-2.39 – 2.17) | -0.63 ± 5.19 | -826.59 |
| 4-HPPA | 24 h | 0.00 (-2.33 – 1.30) | -0.13 ± 3.16 | -2498.59 |
| BA | 0 h | -1.08 (-2.29 – 0.05) | -1.11 ± 1.85 | -167.03 |
| BA | 2 h | 0.99 (-0.60 – 2.58) | 1.21 ± 2.29 | 188.97 |
| BA | 4 h | 4.09 (2.52 – 5.25) | 4.85 ± 5.80 | 119.68 |
| BA | 8 h | 1.52 (0.21 – 3.92) | 1.68 ± 3.01 | 179.36 |
| BA | 24 h | 0.50 (-1.31 – 5.14) | 1.97 ± 4.08 | 206.36 |
| PAA | 0 h | 12.54 (0.03 – 19.6) | 9.43 ± 14.89 | 157.85 |
| PAA | 2 h | 7.86 (-3.58 – 19.22) | 7.74 ± 16.69 | 215.51 |
| PAA | 4 h | 6.17 (-4.36 – 14.63) | 3.74 ± 18.07 | 483.75 |
| PAA | 8 h | -2.34 (-16.35 – 6.03) | -2.66 ± 29.02 | -1088.89 |
| PAA | 24 h | -13.84 (-43.07 – 28.40) | -0.50 ± 53.21 | -10546.05 |

**Supplementary Table S2** List of bins applied in the study covering the spectrum region between δ_H_ 0.5–9.0 ppm (excluding the residual water region, δ_H_ 4.7–5.1 ppm). Chemical shift is calibrated to the internal standard TSP at 0.0 ppm.

| Start of the bin, δ_H_ (ppm) | End of the bin, δ_H_ (ppm) | Name of the annotated metabolites  or not annotated peaks |
| --- | --- | --- |
| -0.02 | 0.02 | TSP |
| 0.7045 | 0.7193 | Cholic acid |
| 0.7193 | 0.7353 | Unknown (Deoxycholic acid) |
| 0.8877 | 0.8943 | Valerate |
| 0.9168 | 0.9234 | Isovalerate |
| 0.9600 | 0.9698 | Leucine |
| 0.9923 | 1.0026 | Valine |
| 1.0138 | 1.0243 | Isoleucine |
| 1.1617 | 1.2100 | Ethanol |
| 1.3328 | 1.3502 | Lactate (+valerate) |
| 1.4685 | 1.4958 | Alanine |
| 1.5613 | 1.5912 | Butyrate |
| 1.9100 | 1.9400 | Acetate |
| 2.2042 | 2.2137 | Propionate |
| 2.2330 | 2.2406 | 5-Aminopentanoate |
| 2.2442 | 2.2465 | Arginine |
| 2.3459 | 2.3608 | Glutamate (+isobutyrate) |
| 2.3851 | 2.3953 | Isobutyrate + Pyroglutamate |
| 2.3979 | 2.4156 | Succinate + Isobutyrate + pyroglutamate |
| 2.6286 | 2.6329 | Methionine |
| 2.7898 | 2.7962 | Aspartate |
| 2.9314 | 2.9448 | Asparagine |
| 3.0059 | 3.012 | One compound between cadaverine, 5-aminopentanoate, and lysine |
| 3.0279 | 3.0355 | Two compounds between cadaverine, 5-aminopentanoate, and lysine |
| 3.0868 | 3.0915 | Cysteine |
| 3.2479 | 3.2608 | Glucose |
| 3.3615 | 3.3676 | Methanol |
| 3.5630 | 3.5688 | Glycine |
| 3.9024 | 3.9085 | Betaine |
| 3.9548 | 3.9663 | Maltose + Serine + Tyrosine |
| 3.9677 | 3.9751 | Serine |
| 4.0376 | 4.0469 | Fructose |
| 4.1400 | 4.1478 | Lactate |
| 4.1483 | 4.1543 | Proline |
| 4.1613 | 4.1728 | Pyroglutamate |
| 4.2400 | 4.2800 | Threonine |
| 5.1800 | 5.2108 | Trehalose |
| 5.3939 | 5.4400 | Maltose |
| 6.5210 | 6.5276 | Fumarate |
| 7.1800 | 7.2114 | Tyrosine + Tryptophan |
| 7.4094 | 7.4612 | Phenylalanine |
| 7.7416 | 7.7536 | Tryptophan |
| 8.4131 | 8.5144 | Formate |

**Supplementary Figure S1** Differential concentrations (FA-treated minus control) of FA and its microbial catabolites over a 24 h time course for each donor (N = 18). 3,4-DHPAA: 3′,4′-dihydroxyphenylacetic acid; 4-HPAA: 4′-hydroxyphenylacetic acid; BA: benzoic acid; PAA: phenyl acetic acid.

**Supplementary Figure S2** Stacked bar plots showing the relative abundance of bacterial phyla across fecal donors.
